# Insecticide resistance status of the *Anopheles funestus* population in Central African Republic: a challenge in the war

**DOI:** 10.1186/s13071-016-1510-9

**Published:** 2016-04-25

**Authors:** Marina Lidwine Olé Sangba, Tanguy Deketramete, Solange Patricia Wango, Mirdad Kazanji, Martin Akogbeto, Mamadou Ousmane Ndiath

**Affiliations:** G4 Malaria Group Institut Pasteur in Bangui, Bangui, Central African Republic; Faculté des Sciences et Techniques, Université d’Abomey Calavi, Cotonou, Bénin; Faculté des Sciences et Techniques, Laboratoire de Biologie Animale Appliquée et de Biodiversité, Université de Bangui, Bangui, Central African Republic; Virology Department, Institut Pasteur in Bangui, Bangui, Central African Republic; Centre de Recherche Entomologique de Cotonou (CREC), Cotonou, 06 BP 2604 Bénin

**Keywords:** Malaria, *Anopheles funestus*, Insecticide resistance, *kdr*, Bangui, Central African Republic

## Abstract

**Background:**

In the Central African Republic, malaria is a major public health problem and the leading cause of death among children. This disease appears to be hyperendemic but no substantial entomological data, including data on *Anopheles* spp. susceptibility to insecticides, is available. This study evaluates, for the first time in the CAR, the status of insecticide resistance in the *Anopheles funestus* population, the second major vector of malaria in Africa.

**Methods:**

WHO standard bioassay susceptibility tests were performed on the *An. funestus* population using F1 generation from gravid females mosquitoes (F0) collected by manual aspirator sampling of households in Gbanikola, Bangui in October 2014 to assess: (i) *An. funestus* susceptibility to bendiocarb, malathion, permethrin, lamda-cyhalothrin, deltamethrin and DDT, and (ii) the effect of pre-exposure to the piperonyl butoxide (PBO) synergist on insecticide susceptibility. Additional tests were conducted to investigate metabolic resistance status (cytochrome P450 monooxygenases, glutathione S-transferases, and esterases).

**Results:**

A high phenotypic resistance of *An. funestus* population to malathion, DDT and pyrethroids was observed with a mortality rate ranging from 23 to 74 %. For the pyrethroid groups, the mortality rate was 35, 31 and 23 % for lambda-cyhalothrin, deltamethrin, and permethrin, respectively. In contrast a 100 % mortality rate to bendiocarb was recorded. Knockdown time (KDT) was long for all pyrethroids, DDT and malathion with KDT_50_ higher than 50 min. Pre-exposure of *An. funestus* to PBO synergist significantly restored susceptibility to all pyrethroids (Fisher's exact test *P* <0.0001) but not in DDT (Fisher's exact test *P* = 0.724). Data from biochemical tests suggest the involvement of cytochrome P450 monooxygenases, esterases and glutatione S-transferases in the resistance of *An. funestus* population from Gbanikola (Wilcoxon test *P* <0.05).

**Conclusion:**

Evidence of biochemical resistance to insecticide was detected in *An. funestus* population from the district of Gbanikola, Bangui. This study suggests that detoxifying enzymes are involved in insecticide resistance of *An. funestus*. However, despite disruptive violence, further research is urgently needed to assess the insecticide susceptibility status of *An. funestus* population in all CAR regions; insecticide resistance could rapidly compromise the success of malaria control programs.

## Background

Despite considerable efforts during the past decade, malaria remains a major public health issue, particularly in Africa [1]. In the Central African Republic (CAR), malaria appears as hyperendemic and in 2013 more than 262,000 people suffering from this disease were treated by Doctors Without Borders teams (MSF) and it also appears to be the cause of a large number of hospital deaths [2]. The mortality rate in the country is generally higher than the “emergency threshold”; this is due to a failing health care system. According to Doctors Without Borders, the CAR has the second lowest life expectancy in the world, i.e. 48 years [3]. In July 2006, the CAR National Malaria Control Programme implemented the first phase of Global Fund Programme for Malaria activities during the period 2006–2009 based on a free LLINs distribution to pregnant women and children under five, with no significant success. Free access to health care and malaria vector control (IRS) are far from being a reality [3–5]. The country is plagued by shortages of essential drugs, logistical constraints and political violence.

Today, there is almost unanimous agreement that the fight against malaria depends largely on an early diagnosis through rapid diagnostic tests (RDTs), cases management by artemisinin-based combination therapy (ACT), combined with vector control using long-lasting insecticidal bednets (LLINs) and indoor residual spraying (IRS) [6]. Pyrethroids are the only group of insecticides currently approved for treating bednets [7, 8], and several studies have demonstrated the efficacy of both (LLINs) and (IRS) for curbing malaria incidence [9–11]. However, these tools are threatened by the emergence of insecticide resistance. Reduced susceptibility to pyrethroids has been confirmed in mosquitoes in west, central, and east Africa and over 60 countries have reported resistance to at least one insecticide and some reported resistance to all insecticide classes (carbamate, organophosphate, pyrethroids and organochlorine) [12, 13], which may contribute to malaria rebound [11, 14]. But in the CAR, no data on the status of malaria vector insecticide resistance are available.

Therefore an entomological study conducted between 2006 and 2010 on the epidemiology of yellow fever, highlighted a specific diversity of *Aedes* and other malaria vectors [15]. The presence of several malaria vectors are recorded with the high prevalence of *Anopheles funestus*, a major vector of malaria throughout much of sub-Saharan Africa*.* The highly anthropophilic and endophilic behaviour of this mosquito makes it an efficient vector of malaria, and in many regions, entomological inoculation rate (EIR) of *An. funestus* far surpasses that of *Anopheles gambiae* [11, 16]. In addition, in several African regions, resistance to insecticides in *An. funestus* population has experienced a rapid increase in recent years [17–21] and represents a threat for malaria control efforts [1].

Recently between October and December 2013, an entomological study of malaria vectors conducted in several districts of Bangui showed the role of *An. funestus* in global malaria transmission including Gbanikola, a district of the capital city Bangui [22]. However, no studies have been conducted so far to investigate the mechanism, level, and types of insecticide resistance in the *An. funestus* population present in the CAR. In this context, we investigated the status of insecticide resistance in *An. funestus* population from Gbanikola and also explored the underling resistance mechanisms. This information will contribute to fill the gap in our knowledge and help to improve future vector control programs in the CAR.

## Methods

### Study area

The CAR is a country of Central Africa with a vast territory of 622,984 km^2^, surrounded by Cameroon to the west, Chad to the north, Sudan and Southern Sudan to the east, and the Democratic Republic of Congo (DRC) and the Republic of Congo to the south. The Ubangi and Mbomou Rivers form most of the southern border with the DRC. The northern part of the country is the high basin of the Chari River. The CAR suffers from frequent flooding due to lack of maintenance of rivers and impressive flow engendered by the rainy season. Most parts of the country are dominated by a tropical climate with a wet season from May to October and a dry season from November to April. Malaria transmission is perennial; all regions of the country are exposed to endemic malaria, with a peak during the rainy season. The district of Gbanikola (N 04°20.436′; E 018°32.213′) is located southeast of Bangui, on the marshy bank of permanent stream. The anopheline larval sites were present all year round. The population was estimated at 4890 inhabitants in 2014. The average annual temperature is around 25 °C and the average rainfall in October (the middle of the rainy season) is 400 mm. During the second week of July 2014, 40 randomly selected households were visited for LLINs coverage. Ownership of bednets in the study population was 66 % and over 70 % of bednets were in a very poor state (holed and ripped).

### Mosquito collection and molecular identification

**Fig. 1:**
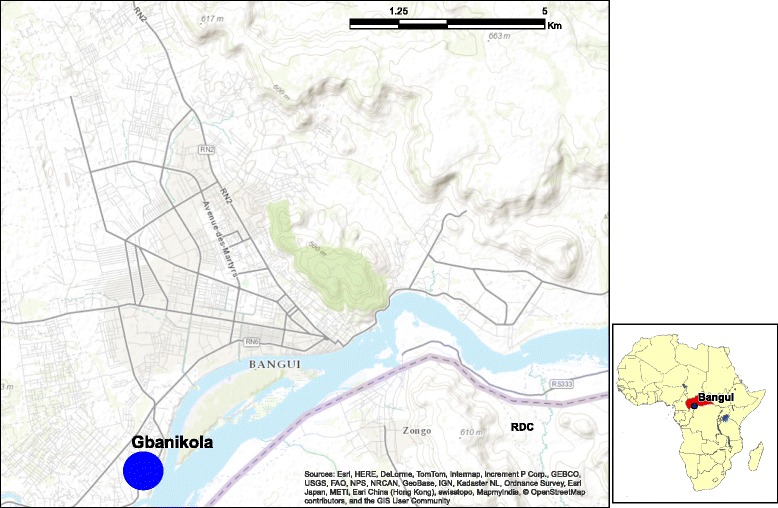
Map of Bangui (Central African Republic) showing study Gbanikola district

Mosquitoes were collected in October 2014 during the rainy season in Gbanikola District, Bangui (Fig. 1). All *Anopheles* females used were morphologically identified as belonging to the *An. funestus* according to the key [23]. Two hundred fifty eight blood-fed *An. funestus* were collected from living rooms at early morning between 6 and 11 am using a manual aspirator and flashlights. The offspring of *An. funestus* females (F0) were pooled and bred under the standard insectary conditions at the insectary of Institut Pasteur of Bangui to retrieve eggs. These eggs were subsequently placed in water under the same conditions. Larvae were fed Tetramin fish food. Pupae were collected and placed in 10 l plastic buckets, which were covered with mosquito gauze with a cotton sleeve for introducing 10 % glucose on filter paper. Adults were maintained at 28 °C, 80 % relative humidity and a 12:12 h light:dark cycle. Unfed 2–3 day females *An. funestus* (F1) were used for insecticide tests. A polymerase chain reaction (PCR) analysis was used to confirm that all F0 females collected were exclusively *An. funestus* [24].

### Susceptibility assays

Bioassays were carried out using WHO test kits for adults mosquitoes [25], to assess the level of sensitivity (or resistance) of mosquitoes to insecticides. Six insecticides of technical grade quality were used: three pyrethroids (permethrin 0.75 %, lambda-cyhalothrin 0.05 %, deltamethrin 0.05 %), one organochlorine (DDT 4 %), one organophosphate (malathion 5 %) and one carbamate (bendiocarb 0.1 %). Impregnated papers were obtained from the WHO reference center (Vector Control Research Unit, University Sains Malaysia, Penang, Malaysia). Four batches of 25 unfed females, aged 2–3 days, were exposed to the diagnostic doses of insecticide treated papers for 60 min at 26 ± 1 °C and 80 % relative humidity. The number of knockdown (KD) mosquitoes was recorded at 10, 15, 20, 30, 40, 50 and 60 min post-exposure. After exposure, mosquitoes were kept in observation tubes and supplied with a 10 % sugar water solution. Expression of final mortality was measured 24 h after exposition. In the absence of *An. funestus* susceptible strain for a positive control, the *An. gambiae* Kisumu strain was used. Mosquitoes exposed to untreated papers were used as the negative control. For each insecticide, a sample of 15 *An. funestus* specimens was randomly selected, including the same number of dead and surviving specimens (when available) and used for molecular tests.

### PBO synergist assays

In the event of confirmed insecticide resistance, additional bioassays were performed using synergist molecule for understanding metabolic processes involved in the detoxification of insecticides. In the case of DDT and pyrethroids, piperonyl butoxide (PBO synergist) was used to inhibit the specific activity of cytochrome P450 (monooxygenases P450). Unfed 2–3 day-old female *An. funestus* (F1) were first exposed for 1 h to 4 % PBO. After PBO exposure, mosquitoes were immediately exposed to deltamethrin 0.05 %, permethrin 0.75 %, lambda-cyhalothrin 0.05 % and DDT 4 % for another hour. For each insecticide, two batches of 25 females were exposed. Another batch exposed only to PBO was used as a control. The number of KD mosquitoes was recorded at 10, 15, 20, 30, 40, 50 and 60 min. Mosquitoes were then transferred into holding tubes and supplied with a 10 % sugar solution. Final mortality was assessed 24 h after exposure. Resistant and susceptible mosquitoes were preserved separately in eppendorf tubes filled with desiccated silica gel and used for molecular tests.

### Biochemical enzyme assays

A total of 78 to 90 F1 *An. funestus* (non-exposed to insecticide) stored at 80 °C were used for biochemical tests. The objective of these tests was to measure the activity of detoxification enzymes revealed by spectrophotometry. Activity levels of cytochrome P450, non-specific esterases (NSE), and glutathione S- transferases (GST) were calculated according the protocol described by Brogdon et al. [26] and by Hemingway et al. [27]. In short, females were homogenized individually in 100 μl of 0.01 M potassium phosphate buffer, pH 7.2, and suspended in 1 ml of buffer. Aliquots of 50 μl were then transferred to microtiter plate wells. Thirty mosquitoes were analyzed per plate in triplicate. Replicates with a variation coefficient that were higher than 0.20 were discarded in order to avoid differences produced by any possible manual errors. All enzymatic reactions were conducted at room temperature. NSE levels were measured at 540 nm after 10 min incubation with 3 mM β-naphthyl acetate. The concentration of the final product (activity per mg of protein) was determined as an endpoint calculated from standard curve of α-naphthyl acetate. For the cytochrome P450 assay, absorbance was measured at 620 nm after 5 min incubation with 2 mM 3,3′,5,5′-tetramethyl-benzidina and corresponding values of enzymatic activity per mg of protein were calculated from a standard curve of cytochrome c. GST levels activity were measured at 340 nm after 5 min incubation in the reaction containing 2 mM reduced glutathione and 1 mM 1-chloro-2,4-dinitrobenzene. An extinction coefficient of 5.76 mM^-1^ (corrected for a path length of 0.6 cm) was used to convert absorbance values to moles of product. GST specific activity was reported as the rate of formation of GSH produced in mmol.min^-1^.mg^-1^ protein. Six negative controls (*An. gambiae* Kisumu strain) per plate were used. Absorbance was measured using a spectrophotometer type “Multiskan FC and Skanit Software” and the adjusted enzymatic mean activity of *An. funestus* was compared to the Kisumu susceptible strain.

### DNA extraction and PCR amplification

DNA was extracted from individual mosquitoes using DNAzol essentially according to the manufacturer’s recommendations (Invitrogen, CA, USA). The total genomic DNA from each mosquito was re-suspended in 100 μl H_2_O and stored at -20 °C until use. All F0 *An. funestus* population were identified by the PCR as described by Cohuet et al. [24]. Briefly, the ITS2 and D3 regions of the rDNA were amplified from 20 ng of template DNA in 50 μl reaction mixture containing 5 μl of 10× reaction buffer (Qiagen), 1.5 mM MgCl_2_, 200 μM each deoxynucleotide triphosphate (Eurogentec), 0.5 units of *Taq* DNA polymerase (Qiagen), and 20 pmol each of forward and reverse primers.

### Data analysis

WHO [25] criteria were used to evaluate the resistance⁄susceptibility status of the tested mosquito populations (98–100 % mortality indicates susceptibility and < 98 % mortality indicates that further investigation is required to confirm resistance). Fifty and 95% knockdown times (respectively KDT_50_ and KDT_95_) were computed with probit regression models. Mortality rates were compared using Fisher's exact test. Biochemical assay data activities (enzymatic activity per mg of protein) of wild specimens of *An. funestus* were compared between the reference strain (*An. gambiae* Kisumu) by Wilcoxon non-parametric and Kruskal-Wallis tests. Statistical analyses were performed using Stata 10.1 software. A *P*-value of 0.05 or less was considered as significant.

### Ethics approval

This study was approved by the National ethics committee of Central African Republic: Authorization No 0101MSANP/CNE2013.

## Results

### *An*o*pheles funestus* insecticide susceptibility

**Fig. 2:**
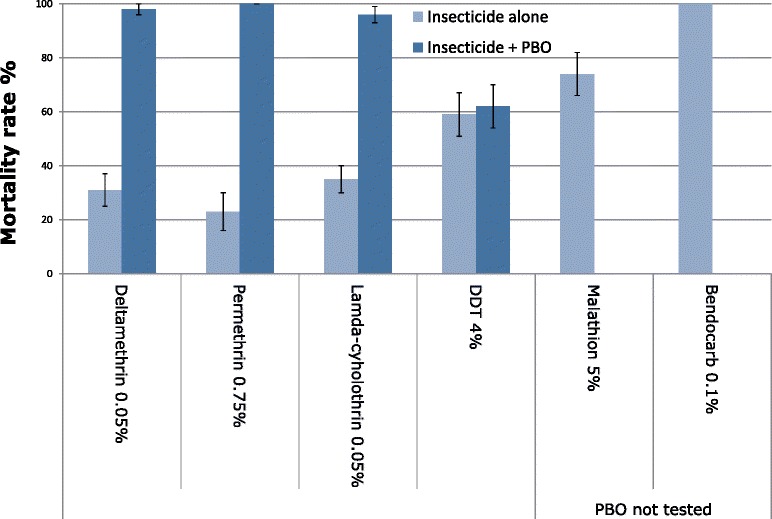
Insecticide bioassay susceptibility test in *An. funestus* population from Gbanikola and effect of piperonyl butoxide (PBO) on the mortality of pyrethroids and DDT 24 h after exposition with 95 % confidence interval (CI)

The mortalities induced by all insecticides on *An. funestus* are illustrated in Fig. 2. The mortality rate of *An. gambiae* Kisumu susceptible strain, used as a control was 100 % for all tested insecticides, thus confirming the quality of the impregnated papers from Malaysia. In the negative control, mortality rates were below 5 %. Based on the WHO criteria, mortality data indicated that mosquitoes were highly resistant to five of the six tested insecticides i.e. malathion, DDT and all pyrethroids (deltamethrin, lambda-cyhalothrin, and permethrin). Mortality rates ranged from 23 to 74 %, which is far below the susceptibility threshold of 98 % (Fig. 2; Table 1). For the pyrethroid groups, the mortality rate after observation time was 35 % (25.7–45.1), 31 % (22.1–41.0), and 23 % (15.1–32.4) for lambda-cyhalothrin, deltamethrin, and permethrin, respectively. The mortality rate was 59 % (48.7–68.7) and 74 % (64.2–82.2) for DDT and malathion respectively. In contrast, mosquitoes were totally susceptible to bendiocarb (Carbamate) with 100 % mortality was observed (Fig. 2; Table 1). Furthermore, 100 % of the population tested was knockdown after 50 min of exposure to bendiocarb with a short KDT_50_ time (23.5 min) (21.8–25.2). Knockdown time was long for the pyrethroid groups (lambda-deltamethrin, lamda-cyhalothrin, permethrin), DDT and malathion with KDT_50_ higher than 50 min (Table 1).

**Table 1:** Bioassay susceptibility tests in *An. funestus* population from Gbanikola

	Mortality %	95 % CI	KDT_50_	95 % CI	KDT_95_	95 % CI
Deltamethrin 0.05 %	31	22.1–41.0	76.2	70.3–82.0	na	–
Lambda-cyhalothrin 0.05 %	35	25.7–45.1	69.4	66.5–72.3	na	–
Permethrin 0.75 %	23	15.1–32.4	71.9	69.0–74.7	na	–
DDT 4 %	59	48.7–68.7	51.7	48.5–54.9	na	–
Malathion 5 %	74	64.2–82.2	61.9	59.8–64.1	na	–
Bendiocarb 0.1 %	100	–	23.5	21.8–25.2	49.0	47.9–50.0

The mortality rate (%) 24 h after exposition, 50 and 95 % knockdown (KDT_50_ KDT_95_) time (min) with 95 % confidence interval (CI), obtained on 100 *An. funestus* for each insecticide tested. na: not applicable

### Effects of PBO synergist on *Anopheles funestus*

Figure 2 shows the insecticidal activities of pyrethroids (permethrin, deltamethrin and lamda-cyhalothrin) and DDT before and after exposition to PBO synergist. Pre-exposure of *An. funestus* to PBO synergist significantly increases the mortality rate in pyrethroid groups. The mean mortality rate ranged from 23, 31 and 35 % before to 100, 98 and 96 % after exposure to permethrin, deltamethrin, and lamda-cyhalothrin, respectively (Fisher's exact test *P* <0.0001). These results indicate the possible role of P450 activities to the resistance of *An. funestus* population. However, no such recovery of susceptibility was observed for DDT as there were no significance difference in mortality rate before (59 %) and after (62 %) PBO exposure (Fisher's exact test *P* = 0.724). In view of these results, it would appear that oxidases (P450) are not the only metabolic resistance process in *An. funestus* population from Gbanikola.

### Biochemical activities of cytochrome P450, esterases and GST

**Fig. 3:**
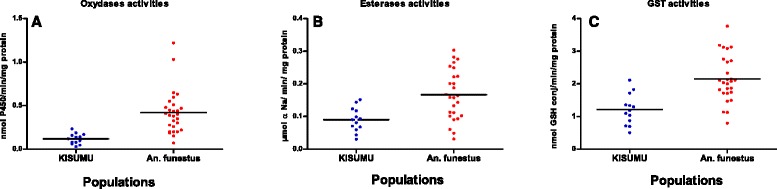
Detoxifying enzyme activities (enzymatic activity per mg of protein) of wild specimens of *An. funestus* population in comparison with *Anopheles gambiae* Kisumu susceptible strain. **a** Cytochrome P450 measured at 620 nm after 5 min incubation; **b** Non-specific esterase (NSE) measured at 540 nm after 10 min incubation; **c** Glutathione S-transferases (GST) measured at 340 nm after 5 min incubation

F1 *An. funestus* females unexposed to insecticides were biochemically analyzed for comparative enzyme activities to *An. gambiae* Kisumu susceptible strain in the absence of *An. funestus* reference susceptible strain. Figure 3 shows the mean level enzymatic activity of cytochrome P450 (a), esterases (b), and GST (c) compared to Kisumu strain. All data from cytochrome P450 showed a significant enzymatic activity (expressed in cytochrome P450 units) compared to the susceptible Kisumu strain reference (Wilcoxon test *P* = 0.0017) (Fig. 3a). A significant correlation between cytochrome P450 enzymatic activity and bioassay mortality data (all pyrethroids) was observed (Kruskal-Wallis test *P* < 0.005) indicating that a metabolic resistance mechanism through the cytochrome P450 genes is operating in the *An. funestus* population from Gbanikola. A significant increased esterase esterases activity (using β-naphthyl acetate) was observed in the *An. funestus* population compared to the Kisumu susceptible strain (Wilcoxon test *P* = 0.006). The mean level enzymatic activity ranged from 0.031 to 0.303 with an average of 0.167 for *An. funestus* while it ranged from 0.03 to 0.151 with an average of 0.09 for the Kisumu susceptible strain (Fig. 3b). A significant increase level of GST activity (using glutathione and 1-chloro-2,4-dinitrobenzene) was observed in the *An. funestus* population from Gbanikola compared to the *An. gambiae* susceptible Kisumu strain (Wilcoxon test *P* = 0.0002). The GST activity in the *An. funestus* population from Gbanikola ranged from 0.79 to 3.77 with an average of 2.15 while it ranged from 0.5 to 2.11 in the Kisumu strain with an average of 1.18 (Fig. 3c).

## Discussion

This study is the first in the CAR to investigate the resistance status of the *Anopheles* population using WHO protocol [25]. The level and type of insecticide resistance present in the *An. funestus* population from Gbanikola, Bangui to six insecticide molecules in WHO diagnostic doses was investigated. Despite the full susceptibility to bendiocarb, the population of *An. funestus* showed a high metabolic resistance to malathion, DDT and pyrethroids (deltamethrin, lamda-cyhalothrin and permethrin). However, although this was the first study done in the CAR, insecticide resistance in wild *An. funestus* populations is not a new phenomenon in Africa. Metabolic resistance of *An. funestus* to pyrethroids and DDT is widespread in the Afrotropical region particularly in Mozambique [17], Uganda [28], southern Africa [29], Benin [18] and Ghana [20].

So far no *kdr* resistance mechanism has yet been found in *An. funestus* [17, 18]. By comparison, this mutation is present in *An. gambiae* population in most parts of Africa and practically in all species (*An. gambiae*, *An. coluzzii*, *An. arabiensis*) and often with both types *kdr-east* and *kdr-west* [13, 21, 30]. The use of pyrethroids and DDT as pesticides for agriculture and for bed-net treatment have both been recognized as factors responsible for the natural selection of resistant mosquitoes in sub-Saharan Africa [31–33]. The emergence and rapid spread of insecticide resistance in *Anopheles* population may impair the effectiveness of malaria vector control based on the use of LLINs and IRS and therefore threaten the sustainability of the current strategies for decreasing malaria transmission [11, 34]. The reduced effectiveness of insecticides coincides with an important international effort to increase bed-net coverage in African countries in order to control malaria transmission [13]. Also, in this study we have not investigated the insecticide resistance origin in Gbanikola district. However, there is high agricultural activity (rice cultivation) in Gbanikola. We believe that the use of pesticides in these fields constitute a possible source of resistance. Nevertheless, resistance in *An. funestus* population, one of the major African malaria vectors, is important enough to advise the national malaria control programme to improve management of insecticide resistance in the *Anopheles* population.

Pre-exposure to PBO synergist has restored the full susceptibility to pyrethroids and not to DDT suggesting the involvement of other metabolic resistance mechanisms beyond that demonstrated by the PBO synergist action. Elsewhere, it has been clearly shown that the metabolic mechanism alone can drive the mosquito resistance to pyrethroid insecticides [18, 35]. However, the effect of PBO synergist is more marked with deltamethrin than with the lamda-cyhalothrin, suggesting the involvement of monooxygenase enzymes [36].

Biochemical analysis measuring the enzymatic activity of cytochrome P450, esterases and GST of the *An. gambiae* Kisumu and *An. funestus* strains showed that cytochrome P450 activity was significantly higher in *An. funestus* than the Kisumu strain. Secondly, there were strong esterases and GST activities in the *An. funestus* population compared to the Kisumu strain. Thus, our data clearly indicate that the pyrethroid resistance in Gbanikola is driven by the involvement of cytochrome P450. Biochemical tests also indicate that GST could be involved in DDT resistance and that esterases could be involved in malathion resistance. Hence this study shows the presence of several types of metabolic resistance mechanisms within the same *An. funestus* population, which is not without consequence in the future development of vector control strategies, especially for targeted malaria control [10, 37]. The combined effect of elevated cytochrome P450 and esterases activity in pyrethroid resistance *Anopheles* mosquitoes has previously been reported [17, 38]. In contrast, the study conducted in Pahou, Benin by Djouaka et al. [18] indicated a high level of insecticide resistance to pyrethroid, DDT, and bendiocarb and total susceptibility to malathion.

Considering our own and previous studies [17, 18, 20, 28, 39], it is clear that the most common resistance mechanism in *An. funestus* is metabolic. This result highlights once again the high variability in insecticide resistance patterns and resistance mechanisms in *An. funestus* population. Pyrethroid and DDT resistance mechanisms are likely not associated with target resistance as no *kdr* mutations associated with resistance to both insecticides was detected. However, further studies are needed to investigate the role of CYP6P9a and CYP6P9b, the two main resistance genes associated with pyrethroid resistance [19].

In terms of vector control research, it is crucial to take into account these different aspects of insecticide resistance. Such resistance has serious implications for malaria control because pyrethroids are the main insecticides recommended by WHO for vector control, especially for bed-net treatment [40, 41]. However the choice of future insecticide in vector control by the CAR national malaria control programme must be reexamined in view of our results.

The bioassays conducted in this study show that *An. funestus* in Gbanikola, Bangui are totally susceptible to bendiocarb which could be a good alternative for IRS in this region. Thereby, in a context marked by a difficult political situation from every point of view, it has added the thorny problem of insecticide resistance. In a country where control structures against malaria exist in name only, it is obvious that substantial efforts must be made by the international community for better management of the issues related to fighting malaria [42].

## Conclusion

For the first time in the CAR, an entomological study has provided data on the insecticide susceptibility status of *An. funestus*. High levels of pyrethroid and DDT resistance were observed in Mbanikola district, and constitute a challenge for resistance management strategies. Therefore, this study provides data to the international community on insecticide resistance in this part of Africa where political, economic and security issues have prevented such research. Despite these difficulties, further research is urgently needed to inventory the insecticide susceptibility status of the *An. funestus* population in all CAR regions including *An. gambiae* (*s.l.*); insecticide resistance could rapidly compromise the successes of malaria control programs throughout the region.
